# From signal overload to shared insight: creating and structuring scientific visuals for comprehension and dialogue

**DOI:** 10.3389/fbinf.2026.1752027

**Published:** 2026-02-13

**Authors:** Joost M. Bakker, Dirma Janse, Martijn van Overbruggen, Maarten C. A. van der Sanden

**Affiliations:** 1 Scicomvisuals BV, Amsterdam, Netherlands; 2 Studio Dirma Janse, Amsterdam, Netherlands; 3 Neurath Visuele Communicatie, The Hague, Netherlands; 4 Ontwerpatelier LUS, Serooskerke, Netherlands

**Keywords:** audiences, comprehension, dialogue, gestalt principles, infographics (information design), presentations, scientific visuals

## Abstract

Effective communication of scientific content can be challenging due to cognitive overload. This is experienced especially during conferences and poster presentations, where the presence of competing stimuli limits message retention. Scientific visuals offer a means to overcome this limitation by emphasizing the essential components of a narrative in a form that is rapidly and intuitively processed. Rather than serving primarily as demonstrations of complexity or markers of personal accomplishment, scientific visuals should function as tools for idea exchange, enabling broader comprehension and facilitating dialogue. The Gestalt principles are an important guide for the visual creation process. These perceptual principles exploit pre-attentive processing mechanisms that allow viewers to extract essential structure and meaning immediately with minimal conscious effort. Effective development of scientific visuals can be approached in three stages: an initial sketch phase, focused on defining the core content and refining the central message, followed by a design phase and refinement phase, in which form, layout and color are used according to perceptual principles. This structured process ensures that complex narratives can be communicated with clarity and precision. By prioritizing cognitive accessibility over ornamental design, visuals become a central and intrinsic component of scientific discourse, supporting insight generation, fostering dialogue, and contributing to collaborative learning and consecutive knowledge building.

## Introduction

People attending multi-day scientific and business conferences often face time-pressured schedules filled with presentations, one-to-one meetings, and poster sessions. In addition, mobile phones demanding constant attention further contribute to unnecessary distraction (Skowronek et al., 2023). Although the human brain can selectively focus on relevant information and flexibly shift attention in dynamic environments (Belledonne et al., 2025), cognitive overload experienced during such events may limit the effective uptake and retention of scientific content (Bawden and Robinson, 2008; Roetzel, 2019). Effective visual representations and information graphics can alleviate cognitive constraints by distilling intricate scientific concepts into accessible, salient formats that facilitate rapid intuitive understanding, enhance memorability, and distinguish key information from surrounding content (Ware, 2008; Healey and Enns, 2012).

When creating scientific visuals for presentation, it is essential to analyze the composition of the audience, as this strongly influences the structure and complexity of the visuals. Conference audiences are typically composed of individuals with varying levels of expertise, commonly falling into three categories (Dingwall et al., 2025).

- The primary audience consists of domain experts, mainly scientists in the presenter’s field, who constitute a small minority. These individuals are likely familiar with the presenter’s work and share a common conceptual framework. They can be very helpful with further deepening and specifying the topic that is presented.
- The secondary audience is formed by scientists from related but distinct fields. This group often represents the majority of attendees and may approach the research from alternative perspectives.
- The hidden audience are often individuals attending out of curiosity, with limited or unrelated expertise. While a minority, they may offer innovative, out-of-the-box insights.

Presenters generally aim to persuade, inform, or entertain their audience, to capture attention and foster engagement, curiosity, and inspiration (Dingwall et al., 2025). However, during preparation many presenters struggle to consider the needs of their audience. In the pursuit of credibility (MacIntosh-Murray, 2007), it may be tempting to fill the presentation with technical depth, publications, or experimental complexity, but neglecting broader accessibility (Pedwell et al., 2017). Such presentations risk alienating all three types of audience, reducing engagement and comprehension. Consequently, much of the communicated information is missed by most, and this is an outcome that should not be acceptable in any presentation. We believe that with careful preparation, presentations can be designed to enhance comprehension across all three types of audiences, fostering dialogue, collaboration, and mutual insight (Corwin et al., 2018; Hutchins, 2020). While refining the design and interpretability of infographics (Guo et al., 2023) undoubtedly enhances understanding, such improvements alone may fall short of actively promoting dialogue and collaborative exchange. We align with Pedwell et al. (2017) in recognizing that audience comprehension is a fundamental objective of poster development. However, we advocate for a broader perspective: presentations should function not merely as vessels for information, but as dynamic platforms for two-way engagement, tools that invite and sustain dialogue rather than simply display. Interestingly, David Bohm, a renowned physicist and philosopher, believed that the strength of dialogue lies in its ability to reveal the hidden assumptions and collective thought patterns that shape our perceptions and interactions. In On Dialogue (Bohm and Nichol, 1996) he defines true dialogue as a collective inquiry into ideas, where the focus shifts from persuasion to shared exploration and learning. According to Bohm, this approach encourages collective knowing and reveals deeper patterns of thought, enabling participants to move beyond individual perspectives. For all types of audience visual design can be a strong entry point to enhance the quality of conversation and hence knowledge building.

Scientific visuals and information graphics that capture the essence of the message while omitting extraneous details that merely showcase expertise can truly have a huge effect on understanding and insight. Interestingly, infographics originally developed to help experts communicate with management also improved experts’ own insight (personal observation). In collaborative design processes, abstract representations such as diagrams or models are often used to integrate distributed knowledge and reveal interdependencies (Alexiou and Zamenopoulos, 2008). This approach is central to managing complexity in collaborative design and is a cornerstone of design thinking, where visual tools help stakeholders articulate and share their understanding of complex systems (Pira et al., 2024). For instance, in a Master course ‘Communication design for innovation’ developed at Delft University of Technology, students were taught to create abstract visuals and logos as part of real-life organizational projects. Through abstraction, they learned to distill the essence of complex problems, facilitating shared understanding among various stakeholders and leading to effective implementation of new strategies through dialogue instead of persuasion (personal observation).

Graphic design is a powerful discipline for visual communication, combining aesthetics and meaning to convey ideas effectively. By arranging visual elements such as typography, color, imagery, layout, and iconography, designers create meaningful structures that guide interpretation. Renowned graphic designers, including Saul Bass, Alan Fletcher, Karel Martens, Malika Favre, and Noma Bar, skillfully apply Gestalt principles (e.g., proximity, similarity, closure, and common region), which describe how humans naturally perceive and group visual elements (reviewed by Wagemans et al., 2012). These principles are grounded in pre-attentive processing, the brain’s ability to rapidly and automatically detect visual features such as color, shape, and position within a few hundred milliseconds (Treisman, 1985; Treisman and Gormican, 1988). This process enables efficient perception and comprehension in complex visual environments and is extensively described in Colin Ware’s Visual Thinking for Design (Ware, 2008).

Leveraging pre-attentive attributes is crucial for effective information visualization. In full agreement with Ware (2008), we propose three fundamental design principles for constructing infographics:

A. Minimize visual clutter by using clear, distinct patterns and a limited color palette.
B. Utilize pre-attentive attributes (e.g., color, size, orientation) to make key patterns immediately recognizable.
C. Organize information hierarchically, grouping related elements and using spatial layout to highlight relationships.

## Creating and constructing an infographic

The design process typically proceeds in three phases (see also Pettersson, 2010):

### Phase 1: content definition

**FIGURE 1:**
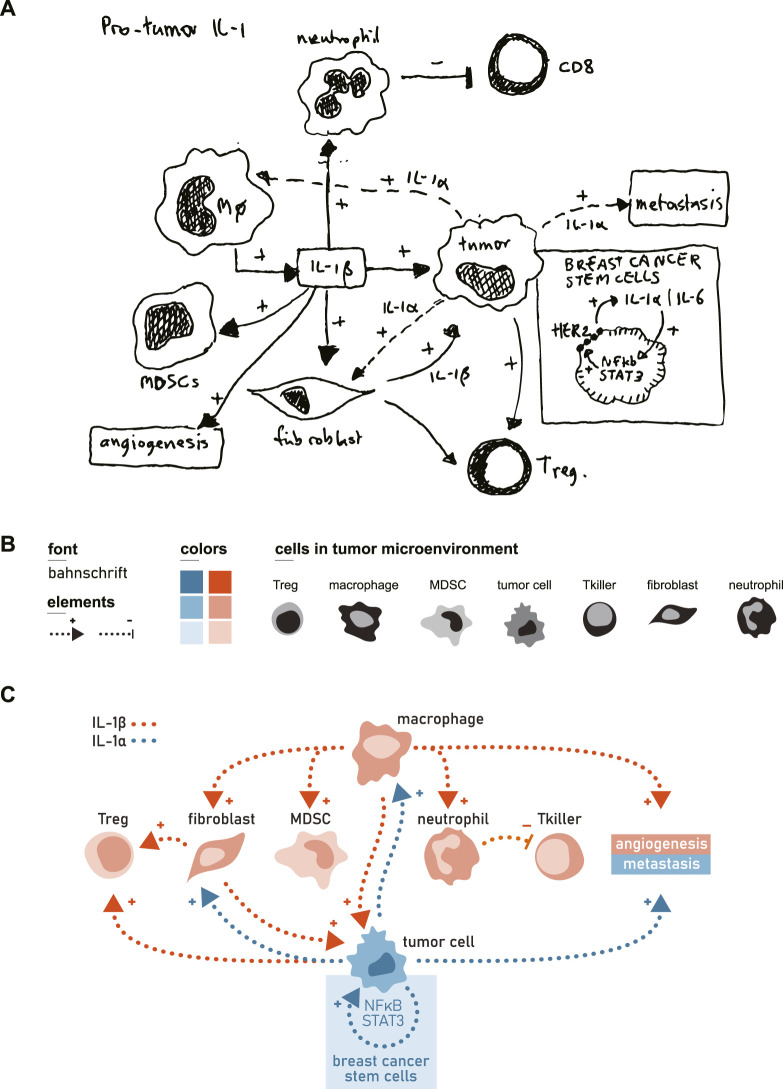
The different phases in creating and constructing an infographic. The aim of this figure was to show the contribution of different cells in the tumor micro-environment with respect to the production of interleukin-1 alpha (IL-1α) and IL-1β, leading to enhanced tumor growth. In addition, a distinction had to be made between tumor cells and tumor-associated stromal cells. The information for this figure was obtained from Baker et al. (2019), Rébé and Ghiringhelli. (2020), Gelfo et al. (2020). **(A)** In phase one of the process, a sketch was developed that showcase the core constituents of the different interactions at play. **(B)** In phase two of the process, the different constituents were visually developed. A font was chosen, which was easy to read and which included Greek characters. Contrasting colors were chosen so that differences were also seen by colorblind viewers. Robust graphic representations of cells were developed with minimal clutter. Arrow lines were dashed to give enough contrast with the other elements. **(C)**. In phase three the complete figure was created, with a clear difference in color and space between the tumor-associated stromal cells in orange shades and the tumor cell in blue shades. The macrophage and the tumor cell, as main producers of IL-1β and IL-1α, were placed at opposite sites to further balance the figure. There was no visual need to use additional colors to discriminate between the IL-1 effects, because the colors of the cells mostly matched the production of both IL-1 family members. The colored quadrant was added to emphasize a feature of IL-1α that seemed to be specific for breast cancer cells. Abbreviations: Treg – regulatory T cell, MDSC – myeloid derived suppressor cell, Tkiller – killer T cell.

Start with defining the core message you want to convey to your audience. Then decide which of the data is truly relevant and help to convey this message. Adopt a critical perspective—ask whether each piece of information is essential to understanding the core message. Another way to find the essence could be to explain the topic to a non-expert, or to condense it into a 1-min summary talk. Decide whether the information is best presented as a single visual representation or as a sequence of panels (e.g., a strip-like format). When you are to present a more abstract subject, use of metaphors is an option too. However, keep in mind that metaphors are inherently imperfect, as mismatches between the source and target concepts are inevitable. While they can clarify complex scientific ideas, they may also impose limiting or misleading frameworks (Taylor and Dewsbury, 2018). Draft an initial sketch of all components (for example, see Figure 1A) and reassess which elements are truly necessary for comprehension and dialogue. Feedback from colleagues at this or the next phases is often invaluable.

### Phase 2: visual creation

In the second phase of the design process, the essential visual elements are created (for example, see Figure 1B). In accordance with the principles of pre-attentive processing, these elements should be clear, distinctive, and free from visual clutter. When multiple illustrations are developed to construct a visual narrative, maintaining design consistency across all figures is essential to ensure coherence and facilitate recognition. The resulting graphical components form a visual language that enables the different types of audience to readily identify patterns and follow the logical progression of the argument. Excessive detail reduces legibility and hinders comprehension; therefore, graphical forms should remain as robust as possible to maximize clarity. The same principle applies to the use of color, which should serve at first instance to enhance understanding and direct attention rather than for purely decorative purposes. Color contrast (luminance) is a key determinant of visual focus and can be used effectively to highlight critical aspects of the presented information.

### Phase 3: structural composition

Arrange the visual in such a way that related information is easily grouped and recognized (Figure 1C), with the aim to quickly guide the viewer through the visual. Use consistent color schemes, spatial alignment, arrows, and minimal text to guide interpretation and emphasize relationships.

## On the use of color

Colors are also conventionally used to represent specific elements, for example, red for blood, green for vegetation, and blue for water. Furthermore, its interpretation might vary across cultural and personal contexts (Kawai et al., 2022) and relate to both positive and negative emotions (Wilson, 1966; Mehta and Zhu, 2009; Chang et al., 2018; Jonauskaitė and Mohr, 2025), thereby further complicating their interpretative use in design.

**FIGURE 2:**
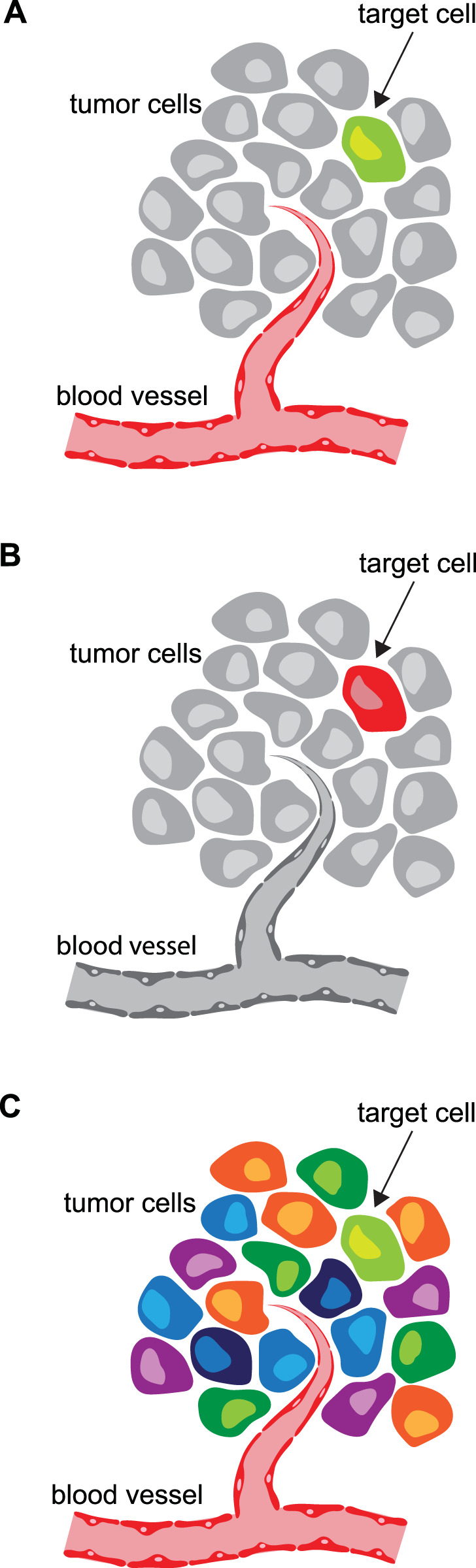
On using colors. This figure of a local vascularized tumor was developed to demonstrate the power of color contrast and luminance to effectively guide attention. **(A)** With the blood vessel in red shades and the target cell in green shades, it can be confusing to quickly focus. **(B)** Following the design principles, the target cell in this example is immediately recognizable. **(C)** A multi-color visualization could make it extremely difficult to focus on the target cell.

However, adhering too rigidly to symbolic or cultural associations may detract from the clarity and focus of the intended message. For example, a bright red blood vessel depicted among mostly grey cells will immediately attract the viewer’s attention (Figure 2A). If the focus should not be on the blood vessel but instead be directed toward the green cell (the target cell in our example), the red blood vessel may distract the viewer. In such cases, assigning a more subdued tone to the blood vessel (or grey in our example) and even using a saturated red to highlight the target cell is more effective (Figure 2B). We believe viewers are well able to analyze colors in relation to their context, and in our example will understand the target cell was made red solely for the purpose of attention. Furthermore, the number of colors employed should be kept to a minimum, as excessive color variation can introduce visual clutter and reduce the overall clarity of the message (Figure 2C). Also, make sure to use colorblind friendly color schemes (Crameri et al., 2020). In agreement with the design principles, color contrast can be used effectively to guide attention, while allowing creative judgment to determine the optimal color scheme for conveying the intended information.

## Conclusion

When scientific visuals are created and constructed to clearly express the essence of complex ideas, they become tools for dialogue rather than mere displays of information. By prioritizing clarity, coherence, and inclusivity over detail and technical sophistication, presenters can bridge gaps between experts and non-specialists alike, enabling diverse audiences to contribute meaningfully to ongoing discussions. They allow participants to grasp relationships, patterns, and processes more intuitively, which in turn foster more informed questions, novel insights, and collaboration between different disciplines. Ultimately, effective visual design in scientific communication helps to transform the presentation from a unidirectional transfer of information into a collective process of gaining insight through comprehension and dialogue. By consciously using design principles grounded in cognitive science and visual perception, presenters can add surplus value to attending scientific conferences.

## Data Availability

The original contributions presented in the study are included in the article/supplementary material, further inquiries can be directed to the corresponding author.
